# Supplementary material for: Modeling glioblastoma heterogeneity as a dynamic network of cell states
Source: Mol Syst Biol. 2021 Sep 16;17(9):e10105. doi: 10.15252/msb.202010105 (PMC8444284; doi:10.15252/msb.202010105)
Supplement: Supplementary file 5 — Source Data for Figure 3 [file MSB-17-e10105-s001.zip › Figure3A_sourcedata/GSEA_3065/nosigdb_state3.GseaPreranked.1622718426028/DOROSZKO_U3180MG_CLUSTER_4.html]

Details for gene set DOROSZKO\_U3180MG\_CLUSTER\_4[GSEA]

|  || Dataset | state3 |
| Phenotype | NoPhenotypeAvailable |
| Upregulated in class | na\_neg |
| GeneSet | DOROSZKO\_U3180MG\_CLUSTER\_4 |
| Enrichment Score (ES) | -0.38595375 |
| Normalized Enrichment Score (NES) | -1.4746262 |
| Nominal p-value | 0.004405286 |
| FDR q-value | 0.062189154 |
| FWER p-Value | 0.889 |
Table: GSEA Results Summary

  

Fig 1: Enrichment plot: DOROSZKO\_U3180MG\_CLUSTER\_4      
 Profile of the Running ES Score & Positions of GeneSet Members on the Rank Ordered List

  

| PROBE | GENE SYMBOL | GENE\_TITLE | RANK IN GENE LIST | RANK METRIC SCORE | RUNNING ES | CORE ENRICHMENT || 1 | PIF1 |  |  | 64 | 0.389 | 0.0097 | No |
| 2 | EMP3 |  |  | 91 | 0.354 | 0.0224 | No |
| 3 | PDGFRA |  |  | 115 | 0.326 | 0.0341 | No |
| 4 | CENPE |  |  | 126 | 0.321 | 0.0471 | No |
| 5 | FKBP2 |  |  | 127 | 0.319 | 0.0612 | No |
| 6 | LBR |  |  | 131 | 0.319 | 0.0750 | No |
| 7 | CNIH4 |  |  | 170 | 0.299 | 0.0838 | No |
| 8 | TXNDC12 |  |  | 186 | 0.292 | 0.0949 | No |
| 9 | DLGAP5 |  |  | 207 | 0.279 | 0.1049 | No |
| 10 | ARL6IP1 |  |  | 210 | 0.278 | 0.1170 | No |
| 11 | ANLN |  |  | 281 | 0.249 | 0.1199 | No |
| 12 | CKAP5 |  |  | 366 | 0.227 | 0.1201 | No |
| 13 | HSPA13 |  |  | 375 | 0.224 | 0.1291 | No |
| 14 | KIF20B |  |  | 411 | 0.216 | 0.1346 | No |
| 15 | KIF11 |  |  | 451 | 0.209 | 0.1393 | No |
| 16 | HMMR |  |  | 478 | 0.202 | 0.1452 | No |
| 17 | SUGP2 |  |  | 490 | 0.200 | 0.1527 | No |
| 18 | ECT2 |  |  | 527 | 0.195 | 0.1572 | No |
| 19 | HNRNPM |  |  | 541 | 0.194 | 0.1642 | No |
| 20 | TMED5 |  |  | 642 | 0.180 | 0.1605 | No |
| 21 | BUB1 |  |  | 717 | 0.168 | 0.1593 | No |
| 22 | EZR |  |  | 759 | 0.162 | 0.1617 | No |
| 23 | KIF14 |  |  | 862 | 0.151 | 0.1565 | No |
| 24 | ACTN1 |  |  | 1079 | 0.132 | 0.1373 | No |
| 25 | HSD17B11 |  |  | 1083 | 0.132 | 0.1427 | No |
| 26 | RBMX |  |  | 1094 | 0.130 | 0.1473 | No |
| 27 | MEX3A |  |  | 1101 | 0.130 | 0.1524 | No |
| 28 | CCNB1 |  |  | 1152 | 0.126 | 0.1521 | No |
| 29 | TTK |  |  | 1177 | 0.123 | 0.1548 | No |
| 30 | GPSM2 |  |  | 1200 | 0.121 | 0.1576 | No |
| 31 | SRRT |  |  | 1256 | 0.118 | 0.1564 | No |
| 32 | SUN2 |  |  | 1275 | 0.117 | 0.1594 | No |
| 33 | GSPT1 |  |  | 1324 | 0.114 | 0.1589 | No |
| 34 | SPAG5 |  |  | 1357 | 0.111 | 0.1601 | No |
| 35 | KIAA0586 |  |  | 1384 | 0.109 | 0.1619 | No |
| 36 | PRR11 |  |  | 1465 | 0.105 | 0.1572 | No |
| 37 | LMO7 |  |  | 1569 | 0.099 | 0.1496 | No |
| 38 | KIF18A |  |  | 1694 | 0.092 | 0.1392 | No |
| 39 | REEP4 |  |  | 1728 | 0.090 | 0.1394 | No |
| 40 | FUBP1 |  |  | 1951 | 0.078 | 0.1170 | No |
| 41 | TMEM160 |  |  | 2072 | 0.073 | 0.1063 | No |
| 42 | BZW1 |  |  | 2121 | 0.071 | 0.1038 | No |
| 43 | ZNF106 |  |  | 2140 | 0.070 | 0.1048 | No |
| 44 | GAS2L3 |  |  | 2174 | 0.069 | 0.1040 | No |
| 45 | CCDC18 |  |  | 2214 | 0.068 | 0.1025 | No |
| 46 | CDCA8 |  |  | 2281 | 0.065 | 0.0977 | No |
| 47 | IDH1 |  |  | 2376 | 0.062 | 0.0895 | No |
| 48 | HNRNPH3 |  |  | 2463 | 0.058 | 0.0820 | No |
| 49 | PSMA4 |  |  | 2605 | 0.053 | 0.0680 | No |
| 50 | USP13 |  |  | 2913 | 0.044 | 0.0342 | No |
| 51 | KNSTRN |  |  | 2976 | 0.042 | 0.0288 | No |
| 52 | RANGAP1 |  |  | 3009 | 0.041 | 0.0269 | No |
| 53 | PCBD2 |  |  | 3173 | 0.036 | 0.0095 | No |
| 54 | RNF26 |  |  | 3209 | 0.035 | 0.0070 | No |
| 55 | TMEM19 |  |  | 3291 | 0.033 | -0.0009 | No |
| 56 | CCT7 |  |  | 3307 | 0.033 | -0.0012 | No |
| 57 | NDE1 |  |  | 3314 | 0.033 | -0.0005 | No |
| 58 | TNFAIP8L1 |  |  | 3349 | 0.032 | -0.0030 | No |
| 59 | RUVBL1 |  |  | 3369 | 0.031 | -0.0038 | No |
| 60 | NCAPD2 |  |  | 3371 | 0.031 | -0.0026 | No |
| 61 | CCNB2 |  |  | 3502 | 0.028 | -0.0165 | No |
| 62 | AURKA |  |  | 3503 | 0.028 | -0.0152 | No |
| 63 | ODF2 |  |  | 3547 | 0.027 | -0.0190 | No |
| 64 | SNAPC1 |  |  | 3686 | 0.023 | -0.0341 | No |
| 65 | SAPCD2 |  |  | 3698 | 0.023 | -0.0344 | No |
| 66 | G2E3 |  |  | 3840 | 0.019 | -0.0499 | No |
| 67 | MRPL47 |  |  | 4072 | 0.013 | -0.0762 | No |
| 68 | ILF2 |  |  | 4112 | 0.012 | -0.0802 | No |
| 69 | MAP4K4 |  |  | 4182 | 0.010 | -0.0878 | No |
| 70 | TROAP |  |  | 4205 | 0.009 | -0.0900 | No |
| 71 | NUP37 |  |  | 4219 | 0.009 | -0.0911 | No |
| 72 | CEP55 |  |  | 4234 | 0.009 | -0.0923 | No |
| 73 | GDI2 |  |  | 4444 | 0.003 | -0.1165 | No |
| 74 | STIP1 |  |  | 4577 | -0.001 | -0.1319 | No |
| 75 | PRMT5 |  |  | 4610 | -0.002 | -0.1355 | No |
| 76 | CEP70 |  |  | 4654 | -0.003 | -0.1404 | No |
| 77 | MDH1 |  |  | 4655 | -0.003 | -0.1403 | No |
| 78 | RUVBL2 |  |  | 4658 | -0.003 | -0.1404 | No |
| 79 | PPP1R35 |  |  | 4690 | -0.003 | -0.1439 | No |
| 80 | SYNCRIP |  |  | 4713 | -0.004 | -0.1463 | No |
| 81 | DCP2 |  |  | 4950 | -0.010 | -0.1733 | No |
| 82 | EEF1AKMT2 |  |  | 5072 | -0.013 | -0.1868 | No |
| 83 | PLK1 |  |  | 5235 | -0.017 | -0.2049 | No |
| 84 | ANP32A |  |  | 5238 | -0.017 | -0.2044 | No |
| 85 | CCT8 |  |  | 5312 | -0.019 | -0.2121 | No |
| 86 | HP1BP3 |  |  | 5330 | -0.019 | -0.2132 | No |
| 87 | SEC22C |  |  | 5339 | -0.019 | -0.2133 | No |
| 88 | CENPA |  |  | 5506 | -0.024 | -0.2316 | No |
| 89 | SPA17 |  |  | 5612 | -0.028 | -0.2426 | No |
| 90 | CDC20 |  |  | 5818 | -0.033 | -0.2649 | No |
| 91 | EIF4E |  |  | 5835 | -0.034 | -0.2653 | No |
| 92 | IMMP1L |  |  | 5866 | -0.035 | -0.2673 | No |
| 93 | MORF4L2 |  |  | 6043 | -0.041 | -0.2859 | No |
| 94 | TIMM9 |  |  | 6180 | -0.046 | -0.2998 | No |
| 95 | RBM42 |  |  | 6195 | -0.046 | -0.2993 | No |
| 96 | KATNA1 |  |  | 6314 | -0.050 | -0.3109 | No |
| 97 | AIMP2 |  |  | 6394 | -0.052 | -0.3178 | No |
| 98 | CDKN2D |  |  | 6491 | -0.056 | -0.3264 | No |
| 99 | SPDL1 |  |  | 6606 | -0.061 | -0.3370 | No |
| 100 | ETV4 |  |  | 6660 | -0.064 | -0.3404 | No |
| 101 | RANGRF |  |  | 6721 | -0.067 | -0.3444 | No |
| 102 | RHNO1 |  |  | 6756 | -0.069 | -0.3453 | No |
| 103 | NUDT11 |  |  | 6775 | -0.070 | -0.3443 | No |
| 104 | ASF1A |  |  | 6818 | -0.072 | -0.3460 | No |
| 105 | KHDRBS1 |  |  | 6868 | -0.074 | -0.3485 | No |
| 106 | CDC25B |  |  | 6935 | -0.078 | -0.3527 | No |
| 107 | PPP2R5C |  |  | 6939 | -0.078 | -0.3496 | No |
| 108 | ECI2 |  |  | 6954 | -0.078 | -0.3478 | No |
| 109 | SEPHS1 |  |  | 7056 | -0.084 | -0.3558 | No |
| 110 | ARPP19 |  |  | 7270 | -0.098 | -0.3763 | No |
| 111 | EMC9 |  |  | 7341 | -0.104 | -0.3798 | No |
| 112 | NUCKS1 |  |  | 7348 | -0.104 | -0.3759 | No |
| 113 | UBALD2 |  |  | 7353 | -0.104 | -0.3718 | No |
| 114 | PSME2 |  |  | 7389 | -0.107 | -0.3711 | No |
| 115 | SDCBP |  |  | 7454 | -0.113 | -0.3736 | No |
| 116 | RAB13 |  |  | 7553 | -0.122 | -0.3796 | No |
| 117 | CDKN2C |  |  | 7609 | -0.128 | -0.3803 | Yes |
| 118 | CRAT |  |  | 7655 | -0.133 | -0.3796 | Yes |
| 119 | EIF4G2 |  |  | 7707 | -0.140 | -0.3793 | Yes |
| 120 | DDAH2 |  |  | 7725 | -0.142 | -0.3750 | Yes |
| 121 | NUDCD2 |  |  | 7755 | -0.147 | -0.3719 | Yes |
| 122 | SMIM14 |  |  | 7774 | -0.148 | -0.3675 | Yes |
| 123 | PAIP1 |  |  | 7795 | -0.151 | -0.3631 | Yes |
| 124 | RAB11A |  |  | 7819 | -0.154 | -0.3590 | Yes |
| 125 | VBP1 |  |  | 7866 | -0.161 | -0.3572 | Yes |
| 126 | MZT1 |  |  | 7891 | -0.164 | -0.3528 | Yes |
| 127 | DAZAP1 |  |  | 7900 | -0.165 | -0.3464 | Yes |
| 128 | RNF5 |  |  | 7933 | -0.173 | -0.3425 | Yes |
| 129 | AAMDC |  |  | 7969 | -0.180 | -0.3386 | Yes |
| 130 | PPP1CC |  |  | 7981 | -0.182 | -0.3318 | Yes |
| 131 | PRR13 |  |  | 7993 | -0.184 | -0.3250 | Yes |
| 132 | CBX1 |  |  | 7995 | -0.184 | -0.3169 | Yes |
| 133 | TMEM256 |  |  | 8056 | -0.194 | -0.3153 | Yes |
| 134 | PLIN3 |  |  | 8092 | -0.202 | -0.3105 | Yes |
| 135 | CIB1 |  |  | 8094 | -0.203 | -0.3016 | Yes |
| 136 | CFAP36 |  |  | 8108 | -0.205 | -0.2941 | Yes |
| 137 | IFT22 |  |  | 8172 | -0.220 | -0.2917 | Yes |
| 138 | CDK2AP2 |  |  | 8181 | -0.222 | -0.2828 | Yes |
| 139 | JPT1 |  |  | 8212 | -0.231 | -0.2760 | Yes |
| 140 | MAP1LC3B |  |  | 8234 | -0.236 | -0.2681 | Yes |
| 141 | TXNDC17 |  |  | 8237 | -0.236 | -0.2578 | Yes |
| 142 | GTF2A2 |  |  | 8261 | -0.243 | -0.2498 | Yes |
| 143 | HDGF |  |  | 8267 | -0.246 | -0.2395 | Yes |
| 144 | COX20 |  |  | 8294 | -0.256 | -0.2312 | Yes |
| 145 | ADI1 |  |  | 8320 | -0.263 | -0.2224 | Yes |
| 146 | SSNA1 |  |  | 8333 | -0.266 | -0.2121 | Yes |
| 147 | FAM120AOS |  |  | 8346 | -0.272 | -0.2015 | Yes |
| 148 | MAZ |  |  | 8350 | -0.273 | -0.1897 | Yes |
| 149 | CALM3 |  |  | 8355 | -0.276 | -0.1780 | Yes |
| 150 | PTTG1 |  |  | 8366 | -0.279 | -0.1668 | Yes |
| 151 | DCTN3 |  |  | 8378 | -0.286 | -0.1555 | Yes |
| 152 | GLRX5 |  |  | 8421 | -0.302 | -0.1470 | Yes |
| 153 | SKA2 |  |  | 8430 | -0.307 | -0.1344 | Yes |
| 154 | NDUFA2 |  |  | 8437 | -0.309 | -0.1214 | Yes |
| 155 | ITGAE |  |  | 8470 | -0.325 | -0.1108 | Yes |
| 156 | COPRS |  |  | 8556 | -0.368 | -0.1044 | Yes |
| 157 | NDUFAF3 |  |  | 8566 | -0.377 | -0.0888 | Yes |
| 158 | TPGS2 |  |  | 8578 | -0.386 | -0.0730 | Yes |
| 159 | UBE2S |  |  | 8655 | -0.458 | -0.0616 | Yes |
| 160 | LGALS1 |  |  | 8744 | -0.718 | -0.0401 | Yes |
| 161 | TMSB10 |  |  | 8749 | -0.922 | 0.0002 | Yes |
Table: GSEA details [plain text format]

  

Fig 2: DOROSZKO\_U3180MG\_CLUSTER\_4: Random ES distribution      
 Gene set null distribution of ES for **DOROSZKO\_U3180MG\_CLUSTER\_4**
